# Effect of rapid weight loss on mood states and burnout of Brazilian jiu-jitsu athletes during a competitive process

**DOI:** 10.3389/fpsyg.2023.1114816

**Published:** 2023-05-02

**Authors:** João Carlos Alves Bueno, Rodrigo Batalha Silva, Pierluigi Diotaiuti, Leonardo Vidal Andreato, Alexandro Andrade

**Affiliations:** ^1^Laboratory of Sport and Exercise Psychology, Center of Health and Sport Science, Santa Catarina State University, Santa Catarina, Brazil; ^2^Department of Human Sciences, Society and Health, University of Cassino and Southern Lazio, Cassino, Italy; ^3^School of Health Sciences, University of the State of Amazonas, Manaus, Brazil

**Keywords:** martial arts, combat sports, performance, sport psychology (SC49365), weigh loss

## Abstract

The current study aimed to analyze the psychological alterations resulting from rapid weight loss in the mood states and burnout of male Brazilian jiu-jitsu athletes. For the purpose of this study, 31 Brazilian jiu-jitsu athletes participated, divided into two groups (RWLG: rapid weight loss group and CG: control group). Data collections were performed at three moments (1: baseline, before weight loss; 2: weigh-in, during the official competition; and 3: recovery, 7 to 10 days after the competition), with application of the Brunel Mood Scale (BRUMS) and Athlete Burnout Questionnaire (ABQ). Considering the outcomes, for body mass, the athletes from RWLG presented mean reductions of 3.5 kg (4.2% of the initial body mass). For mood states, both groups (RWLG and CG) presented a moment effect for tension and confusion, with higher levels during weigh-in compared to baseline and recovery (*p* < 0.05). For burnout, both groups showed low levels of burnout, with no effect of moment, group, or interaction in the analyses (*p* > 0.05). Based on these results, it is concluded that weight loss, in the magnitude performed in this study, did not generate an additional impact on mood or level of burnout in Brazilian jiu-jitsu athletes during a competitive process.

## Introduction

Brazilian jiu-jitsu is a combat sport that has been growing in popularity around the world, with championships that bring together both amateur practitioners and professional athletes (Scoggin et al., 2014). One of the characteristics of Brazilian jiu-jitsu competitors is that they undergo a large amount of body mass loss over a small number of days before they compete (Brito et al., 2012; White and Kirk, 2021), representing an attempt for athletes to gain a physical advantage when competing against lighter opponents (Barley et al., 2019). In Brazilian jiu-jitsu, previous studies identified that between 57% (Brito et al., 2012) and 88% of male competitors reduced body mass in order to compete in a category with a lower body mass limit (Bueno et al., 2023).

This process of weight loss prior to competing is very common in combat sports, with a high prevalence of body mass reduction being observed among fighters of different modalities (Drid et al., 2021; Ranisavljev et al., 2022; Roklicer et al., 2022) and occurring at virtually all competitive levels (Artioli et al., 2010). To achieve this goal, athletes adopt the most varied methods, including even methods that are highly dangerous to health (e.g., use of laxatives, diuretics, diet pills, and vomiting) (Drid et al., 2021).

However, there is a consensus that for an athlete to attain good competitive results, it is not only necessary to have good performance in physical abilities and skills, but also to have well-prepared mental abilities (Brandt et al., 2021). Research has shown that emotional states can influence an athlete’s sports performance (Allen and De Jong, 2006; Jensen et al., 2013; Massey et al., 2013), thus demonstrating the importance of mental skills for high-level sports performance (Devonport, 2006; Bertollo et al., 2009; Hatzigeorgiadis et al., 2009).

In this sense, one of the psychological aspects addressed by recent research in combat sports athletes is mood states (Brandt et al., 2018; Maynard et al., 2018; Andrade et al., 2019). Mood is an emotional state with a short-lived characteristic, that can reflect feelings such as anxiety, happiness, and sadness, among others (Andrade et al., 2019). Mood can vary in intensity and duration and involves five negative factors (anger, tension, depression, fatigue, and confusion) and one positive factor (vigor) (Rohlfs et al., 2008). Although some studies investigating the effect of weight loss on the mood states of combat sport athletes showed changes, such as increased anger, tension, depression, fatigue, and decreased vigor among judo athletes (Fortes et al., 2018), or increased confusion among wrestlers (Marttinen et al., 2011), a gradual diet did not promote changes in the mood profile (Maynard et al., 2018). Furthermore, there is no consensus on whether there are associations between changes in mood profile resulting from weight loss and physical performance (Hall, 2001; Degoutte et al., 2006; Marttinen et al., 2011; Lakicevic et al., 2020).

Another psychological aspect to be contemplated for combat sports athletes in general is burnout, which is considered a multidimensional construct and manifests itself in athletes through three dimensions: physical and emotional exhaustion; devaluation; and a reduced sense of accomplishment (Smith, 1986; Raedeke, 1997; Raedeke and Smith, 2001). Due to burnout, professional athletes may present a decrease in their performance, reaching the point of permanent abandonment of the sport. The feeling of exhaustion, accompanied by negative thoughts and regrets about participating in the sport, can lead the individual to avoid practicing the modality after abandonment, even for fun or health (Gustafsson et al., 2018).

Due to the high load of physical training and high emotional demand that Brazilian jiu-jitsu athletes are submitted to Campos et al. (2022), as well as the repeated practices of rapid weight loss that they perform (White and Kirk, 2021), it is not known for sure how much this set of factors can impact the emergence of burnout in this population. In addition, no studies were found that investigated burnout in combat sports athletes undergoing rapid weight loss, evidencing a gap in the literature. Furthermore, although some studies have investigated the effects of weight loss on mood in other combat sports (Finn et al., 2004; Marttinen et al., 2011; Fortes et al., 2018; Isacco et al., 2020), little is specifically known about Brazilian jiu-jitsu (Maynard et al., 2018).

Therefore, the objective of this study was to analyze the psychological changes resulting from rapid weight loss in the mood and burnout of Brazilian jiu-jitsu athletes.

## Methods

### Experimental design

This research is characterized as a non-randomized clinical trial. For the recruitment of participants, Brazilian jiu-jitsu coaches were contacted to authorize data collection with their teams of competitors. After receiving this authorization, the athletes were invited to participate in the study, and an explanation of the objectives and procedures that would be carried out was provided. The volunteers were divided into the control group (CG), consisting of athletes who would not undergo weight loss; and the rapid weight loss group (RWLG), consisting of athletes who would undergo weight loss before competing. The group definition followed the strategies (loss or no weight loss) defined by the athletes and coaches, without any interference from the researchers. All athletes were evaluated at three moments. The first moment (baseline), for WLG athletes, was performed ~7 days before the weigh-in, and for CG athletes, it was established 14 days before the official weigh-in. At this first moment, questionnaires on the athletes’ general characteristics, the Brunel Mood Scale (BRUMS), and the Athlete Burnout Questionnaire (ABQ) were applied. The second moment (weigh-in) was carried out on the day of the official weigh-in of the athletes; and the third moment (follow-up) was performed 7 to 10 days after the competition. At the second and third moments, both groups completed the BRUMS and ABQ, as well as self-reporting body mass information. At the three moments of collection, the questionnaire instruments were applied in an appropriate place so that they could be completed with as little interference as possible. Two researchers were present at the collection site, to clarify the research objectives and any possible doubts regarding the completion of the instruments.

### Participants

Thirty-one Brazilian jiu-jitsu male athletes, with a minimum graduation of blue belt, were volunteers in this research. All participants had competitive experience at national and international levels.

Regarding the level of education among the investigated athletes, there was a higher frequency of high school for both the CG (n = 12; 60%) and RWLG (n = 6; 55%). For another type of profession, both the CG (n = 13; 65%) and RWLG (n = 9; 82%) reported having another source of income. The most frequent graduation level between the groups in both groups (CG: n = 14 70%; RWLG: n = 4; 36%) was the black belt. Both the CG (n = 14; 70%) and RWLG (n = 8; 73%) reported not having any type of investment/sponsorship as financial support.

The inclusion criteria to participate in the study were; aged between 18 and 40 years, having a minimum blue belt graduation, having been training and competing regularly for at least 2 years, and not having an injury or health problem at the beginning of the study that could interfere with the results. The exclusion criteria adopted were the use of prohibited substances or mood-regulating drugs.

After receiving an explanation about the research objectives, all participants signed an informed consent form. This study was approved by the Human Research Ethics Committee of the State University of Santa Catarina (UDESC) under protocol n. 2.776.490.

### Instruments

#### Questionnaire for the general characterization of athletes in combat sports

The Questionnaire for the general characterization of athletes in combat sports modalities was adopted, which has been used in previous studies (Brandt et al., 2015), and includes data on weight, height, age, sex, time of BJJ practice, and BJJ graduation, arranged in open (weight, height) and closed questions.

#### Brunel mood scale

The BRUMS is a short adapted version of the Profile of Mood States – POMS (McNair et al., 1971) which allows quick measurement of the mood state, through 24 items of the scale comprising the following six subscales: anger, confusion, depression, fatigue, tension, and vigor. Each subscale contains four items, with the sum of responses ranging from 0 to 16 (Rohlfs et al., 2008). The validation study showed internal consistency with Cronbach’s alpha values greater than 0.76 (Rohlfs et al., 2008).

#### Athlete burnout questionnaire

The QBA is the Brazilian version of the Athlete Burnout Questionnaire – *ABQ* (Raedeke and Smith, 2001), translated and validated for use in Brazilian athletes (Pires et al., 2006). The questionnaire consists of 15 statements that correspond to feelings related to burnout. For each statement, the response is positioned on a Likert-type scale ranging from 1 (almost never), 2 (rarely), 3 (sometimes), 4 (often), and 5 (almost always). Each statement refers to a construct related to the manifestation of burnout in athletes: physical and emotional exhaustion (EFE); reduced sense of accomplishment (RSR); and sporting devaluation (DES). In relation to general burnout, the results are attributed from the arithmetic mean of the 15 items of the instrument (Raedeke, 1997). The internal consistency results of the ABQ validation study showed a Cronbach’s alpha value of 0.82 (Pires et al., 2006).

### Statistical analysis

Data are presented as mean and standard deviation. For the mood and burnout profile variables, the two-way analysis of variance (ANOVA) model (group x moment) was applied, with repeated measures in the second factor, using the Bonferroni *post hoc* when a significant difference was found. The Mauchly test was applied to verify the sphericity of the data, and the Greenhouse–Geisser correction was used when necessary. Additionally, the magnitude of the differences was evaluated using the effect size, calculated (eta squared, η2) and interpreted as follows: < 0.2 (small), > 0.2 and < 0.8 (moderate), and > 0.8 (large) (Cohen, 1988). Significance was set at 5%. Data were analyzed using the software Statistical Package for the Social Sciences (SPSS), version 20^®^.

### Results

At baseline, the investigated athletes had an average age of 29.61 (±7.11) years, body mass of 84.27 (±15.05) kg, BJJ practice time of 17.06 (±5.81) years, and weekly training of 12.67 (±13.89) hours. The BJJ graduation of the athletes in the sample consisted of 8 (25.80%) blue belts, 4 (12.91%) purple belts, 1 (3.22%) brown belt, and 18 (58.07%) black belts.

RWLG lost an average of 3.4 ± 2.4 kg (range 1–10 kg) and the magnitude of weight loss was 4.18%. The mean weight regained at follow-up for all athletes was 2.3 ± 2.5 kg and for RWLG it was 4.1 ± 2.1 kg. A moment effect was found (F_2,__58_ = 18.046; *p* < 0.001) with a significant difference between baseline weight and the moment of weigh-in (*p* = 0.001) and between weigh-in and follow-up (p < 0.001). An interaction effect was also found (F_2,__58_ = 14.457; *p* < 0.001) between moments and groups. The *post hoc* test identified lower values for RWLG at weigh-in compared to baseline (*p* < 0.001) and at weigh-in compared to recovery (*p* < 0.001). No group effect was found (*p* = 0.295) (Table 1).

**Table 1:** Body mass (kg) in the investigated athletes at the collection moments.

	Baseline^b^	Weigh-in^b^^,^^c^	Recovery^c^
Control group (*n* = 11)	87.5 ± 18.6	87.6 ± 18.5	88.1 ± 19.3
Rapid weight loss group (*n* = 20)	82.5 ± 12.9^d^	79.1 ± 12.9^d^^,^^e^	83.2 ± 13.8^e^

Data expressed as mean ± standard deviation.

a Interaction effect.

b Baseline moment × weigh-in (*p* < 0.05).

c Weigh-in × recovery (*p* < 0.05).

d Baseline × weigh-in (*p* < 0.05).

e Weigh-in vs recovery (*p* < 0.05).

Data on the mood of the investigated athletes are described in Table 2.

**Table 2:** Mood states of combat sports athletes submitted or not to weight loss to compete.

	Control group (*n* = 11)			Rapid weight loss group (*n* = 20)			*F*-value		
	Baseline	Weigh-in	Recovery	Baseline	Weigh-in	Recovery	Group	Moment	Interaction
Tension^a^	3.64 ± 3.88^b^	7.27 ± 2.86^b^^,^^c^	2.18 ± 3.73^c^	4.10 ± 2.73	5.55 ± 3.80	2.90 ± 3.23	0.03	13.60	1.58
Depression	0.55 ± 1.29	0.91 ± 1.64	0.36 ± 0.67	1.00 ± 2.08	1.15 ± 2.32	1.50 ± 2.98	0.88	0.19	0.63
Anger	2.00 ± 3.68	2.45 ± 3.61	1.64 ± 3.66	3.65 ± 4.25	5.00 ± 4.07	3.25 ± 4.20	2.36	1.72	0.27
Vigor	10.09 ± 2.79	10.64 ± 2.87	9.18 ± 2.75	11.30 ± 2.81	11.30 ± 3.01	10.40 ± 3.02	1.32	2.48	0.16
Fatigue	4.36 ± 5.44	5.27 ± 4.86	4.82 ± 5.17	4.75 ± 5.01	5.85 ± 4.51	5.55 ± 4.66	0.11	1.31	0.03
Confusion^a^	2.82 ± 5.36^b^	4.64 ± 5.39^b^^,^^c^	1.82 ± 4.77^c^	1.15 ± 1.73	1.90 ± 2.59	0.90 ± 1.52	2.26	7.56	1.66

Data expressed as mean ± standard deviation.

a Moment effect (*p* < 0.05).

b Baseline × weigh-in (*p* < 0.05).

c Weigh-in × recovery (*p* < 0.05).

For tension, there was a moment effect (F_1.397,__40.510_ = 13.60; p < 0.001; *η^2^* = 0.31, moderate), with higher values at weigh-in compared to baseline (*p* = 0.01) and follow-up (*p* = 0.025). However, there was no group (*p* = 0.847) or interaction effect (*p* = 0.220). The *post hoc* test identified higher tension values for the CG at weigh-in compared to baseline (*p* = 0.033) and at weigh-in compared to recovery (*p* = 0.015).

For confusion, there was a moment effect (F_2, 58_ = 7.56; p = 0.001; *η^2^* = 0.20, small), with higher values at weigh-in compared to baseline (*p* = 0.014) and follow-up (*p* = 0.008). However, there was no group (*p* = 0.143) or interaction effect (*p* = 0.198). The *post hoc* test identified higher stress values for the CG at weigh-in compared to baseline (*p* = 0.024) and weigh-in compared to recovery (*p* = 0.006).

For anger, there was no group (*p* = 0.135), moment (*p* = 0.186), or interaction effect (*p* = 0.759). For vigor, there was no group (*p* = 0.259), moment (*p* = 0.104), or interaction effect (*p* = 0.806). For depression there was no group (*p* = 0.354), moment (*p* = 0.770), or interaction effect (*p* = 0.499). For fatigue, there was no group (*p* = 0.740), moment (*p* = 0.276) or interaction effect (*p* = 0.962).

Data on burnout had a sample loss of 10 athletes. The data for the investigated athletes are presented in Table 3.

**Table 3:** Values of burnout components of combat sports athletes submitted or not to weight loss to compete.

	Control group (*n* = 11)			Rapid weight loss group (*n* = 14)			*F*-value		
	Baseline	Weigh-in	Recovery	Baseline	Weigh-in	Recovery	Group	Moment	Interaction
RSR	2.40 ± 0.30	2.43 ± 0.40	2.32 ± 0.28	2.69 ± 0.65	2.83 ± 0.60	2.70 ± 0.67	2.89	3.02	0.63
EFE	1.58 ± 0.50	1.54 ± 0.41	1.45 ± 0.36	1.67 ± 0.96	1.89 ± 0.96	1.74 ± 0.93	0.65	1,18	1,38
DES	2.01 ± 2.04	1.50 ± 0.55	1.40 ± 0.37	1.91 ± 1.30	1.69 ± 0.76	1.69 ± 0.75	1.15	1.41	0.27
BG	2.00 ± 0.72	1.83 ± 0.37	1.72 ± 0.22	2.09 ± 0.76	2.13 ± 0.65	2.04 ± 0.68	1.12	1.46	0.89

Data expressed as mean ± standard deviation. RSR, Reduced sense of accomplishment; EFE, Physical and emotional exhaustion; DES, Sports devaluation; BG, General burnout.

For reduced sense of accomplishment (RSR), physical and emotional exhaustion (EFE), sports devaluation (DES), and general burnout (BG), no group (RSR: *p* = 0.103; EFE: *p* = 0.426; DES: *p* = 0.701; BG: *p* = 0.300), moment (RSR: *p* = 0.072; EFE: *p* = 0.308; DES: *p* = 0.248; BG: *p* = 0.243), or interaction effects were found (RSR: *p* = 0.501; EFE: p = 0.259; DES: *p* = 0.631; BG: *p* = 0.379).

## Discussion

The aim of the present study was to analyze the psychological changes resulting from rapid weight loss in mood and burnout of Brazilian jiu-jitsu athletes. The results found showed that the athletes submitted to weight loss presented an average decrease in body mass of 3.4 kg, with a magnitude of weight loss of 4.3%. A greater difference in weight was found between the moment of weigh-in and the moment of baseline and recovery. For mood, a moment effect was found for tension and confusion, with higher values at the weigh-in compared to the other moments. For burnout, no significant difference was found in moment, group, or interaction.

For the mood results, an effect was observed for the moment, with an increase in tension between the weigh-in and the beginning of weight loss, corroborating other studies with wrestling, Brazilian jiu-jitsu, MMA, and Muay-Thai athletes (Marttinen et al., 2011; Maynard et al., 2018; Nascimento-Carvalho et al., 2018). There was no difference in tension in athletes who lost weight in the present research, a fact that corroborates studies that showed high tension after weight loss among judo athletes (Filaire et al., 2001; Degoutte et al., 2006; Yoshioka et al., 2006; Koral and Dosseville, 2009). In these studies, values for weight loss and magnitude of weight loss were verified, ranging from 3 to 5 kg of weight loss and 3 to 5.6% of body mass loss. However, considering the athletes who lost weight in the present research, even with weight loss data similar to the previous studies, there did not seem to be an influence on the athletes’ tension.

The increase in tension at the weigh-in moment may have been influenced by the results of athletes who did not lose weight, who showed a greater percentage increase (99.7% vs. 35.4%). In this sense, an increase in tension was also observed in other studies in Brazilian jiu-jitsu athletes (Brandt et al., 2015; Maynard et al., 2018; Andrade et al., 2019). This high tension may not be a result of weight loss, but could be associated with the tension and anxiety generated by the proximity of the competition (Andreato et al., 2014). Care should be taken with tension at moderate or high levels, as this can lead to musculoskeletal tension that may be related to a higher incidence of injuries, due to diminished mental and physical capacity (Andrade et al., 2016).

No differences in confusion were observed in athletes who lost weight in the present study, thus differing from other studies with combat sports athletes (Barley et al., 2018), judo (Filaire et al., 2001; Koral and Dosseville, 2009), MMA (Brandt et al., 2018), and wrestling (Marttinen et al., 2011). Furthermore, high levels of confusion showed a positive correlation with the magnitude of weight loss in amateur boxers (Hall, 2001). The result of a higher level of confusion during the weigh-in compared to baseline and recovery in athletes who did not lose weight was not found in any similar study and this may have influenced the moment effect observed in the current study. These outcomes demand attention, as confusion is characterized by feelings of uncertainty, bewilderment, and emotional instability (Andrade et al., 2019), and at high levels can generate psychological stress (Yoshioka et al., 2006) and impair the performance of fighters (Hall, 2001).

The weight loss that the RWLG underwent was insufficient to cause acute changes in anger. These results corroborate studies in judo (Yoshioka et al., 2006; Koral and Dosseville, 2009) and MMA (Coswig et al., 2019), while one study found greater anger in a group of MMA athletes who did not lose weight to compete (Brandt et al., 2018). However, other studies have found an increase in anger as a result of weight loss in different modalities of combat sports (Filaire et al., 2001; Hall, 2001; Degoutte et al., 2006; Fortes et al., 2018; Nascimento-Carvalho et al., 2018). In addition, a positive correlation was found between anger and the amount of weight lost in adolescent wrestling athletes from two categories (Karninčić et al., 2016).

Changes in anger may be an important variable when it comes to combat sports. Anger at moderate levels can have a facilitating effect to increase vigor levels and may be associated with the athlete’s level of activation (Brandt et al., 2015). However, the literature does not show whether heightened anger could be a characteristic between winning and losing in fighters (Wong et al., 2006; Andrade et al., 2019; Isacco et al., 2020). Further studies, with larger numbers of athletes, and of different sexes are needed for better understanding of the effects of anger in fighters.

In the present study, no difference was found in vigor for moment or group interaction. A similar result was reported in the study of Brandt et al. (2018), where no differences were observed in the vigor of MMA athletes who lost and did not lose weight. In the opposite direction, some studies show worsening vigor as a result of weight loss in athletes from different combat sports (judo, MMA, boxing, *wrestling*) (Filaire et al., 2001; Hall, 2001; Degoutte et al., 2006; Yoshioka et al., 2006; Koral and Dosseville, 2009; Marttinen et al., 2011; Fortes et al., 2018; Nascimento-Carvalho et al., 2018). However, in the study of Karninčić et al. (2016), no correlation was found between the amount and magnitude of weight lost and the athletes’ vigor. Decreased vigor seems to impair athletes’ perception and performance. In a study of Olympic athletes who lost weight to compete, it was found that 45% felt tired, 35% felt weaker, and 32.5% felt more irritable and exhausted (Abreu et al., 2015). More studies, with a greater number of athletes are needed to better evidence the effect of weight loss on the vigor of combat sports athletes.

One reason that may explain the non-alteration in mood states of Brazilian jiu-jitsu athletes who lost weight is the fact that, in most Brazilian jiu-jitsu competitions, athletes undergo the official weigh-in minutes before competing (IBJJF. Rule Book, 2021), giving the athletes time to look for ways and methods of weight loss with less potential for danger to the body. Another explanation for this result can be found through the extensive practice time found in the athletes of the present research. The experience that the athlete accumulates due to the different moments of stress in the weight changes that occur during their career, can generate physical and emotional adaptations, causing the athlete to display better emotional regulation, despite water and nutritional deficits (Brandt et al., 2018).

Thus, although the weight loss process did not generate expressive mood disturbances for Brazilian jiu-jitsu athletes, monitoring the mood state of these athletes during the preparation process for competitions is essential, especially since a recent systematic review identified that the mood state components seem to be the main psychological parameters that can affect the performance of judo athletes, with the most successful athletes likely possessing higher vigor and good control of negative aspects of mood state (Rossi et al., 2022).

Considering burnout, in general, the athletes in the present study had low scores, similar to other individual sports, such as tennis (Casagrande et al., 2014, 2017), and other contact sports, such as rugby (Cresswell and Eklund, 2005; Hodge et al., 2008). In sport psychology, it has been argued that burnout is more common in individual sports than in team sports, due to the higher levels of strength and time demands required (Smith, 1986; Coakley, 1992; Diotaiuti et al., 2021a). However, in the study of Gustafsson et al. (2007), higher burnout was found in male team sports athletes on the physical and emotional exhaustion and sports devaluation subscales, although the moderate to low effect size limits the scope of inferences.

Traditionally, training stress and harsh practice conditions have been identified as the main causes of burnout (Silva, 1990; Vealey et al., 1998; Konig et al., 2001), corroborating previous explanations of the supposed higher prevalence of burnout in athletes of individual modalities (Gustafsson et al., 2007). These findings should alert researchers, athletes, and coaches, as in many combat sports, athletes undergo training with high volumes and intensity (Andrade et al., 2019; Campos et al., 2022) and, in addition to the various weight loss methods, these can be factors that cause burnout symptoms in fighters.

Regarding the investigation of burnout in combat sports, there are still few studies (Bicalho and Costa, 2018). In the study of Gaia et al. (2020), when investigating burnout in Brazilian jiu-jitsu, karate, boxing, and Muay Thai athletes, a low level of burnout was observed in the three dimensions, with a lower number for the EFE. In the Katkat study (Katkat, 2015) with judo, karate, and taekwondo athletes, moderate to high levels were found in the reduced sense of accomplishment and sports devaluation scales and high levels in the physical and emotional exhaustion scale. In both studies, data collection was transversal, without observing or controlling the influence of a possible interference of weight loss on the athletes’ data.

The low levels of burnout in these combat sports athletes may be indicative of better adaptation to the physical and psychological demands of the modalities and a balance between training stress and recovery (Pires et al., 2019). In addition, psychological skills training and the use of self-regulation and coping strategies are practices that help combat sports athletes to withstand stressful events more easily (Belem et al., 2016; Diotaiuti et al., 2021b). As the sample of fighters in the present research reported an average of approximately 17 years of practice, this may be an indication of the greater experience of athletes when facing recurrent stressful events (Gaia et al., 2020).

Despite the contributions of this study, these findings should consider the research limitations: lack of direct assessment of body mass, control of the strategies used for weight loss, monitoring of the training load during the preparation process, and physical performance indicators of performance and recovery. Finally, it is suggested that future studies investigate the influence of the repeated practice of weight loss on mood states, burnout, and other psychological variables of fight modality athletes, in addition to studying female athletes.

## Conclusion

Based on these results, weight loss, in the magnitude performed in this study, did not generate any additional impact on mood or level of burnout in Brazilian jiu-jitsu athletes during a competitive process. For mood, a moment effect was found for tension and confusion, with higher values at the weigh-in compared to the other moments; but without a group or interaction effect. The burnout of the investigated athletes was low in both groups and no significant differences were found for moments, groups, or interactions.

## Data availability statement

The original contributions presented in the study are included in the article/supplementary material, further inquiries can be directed to the corresponding author.

## Ethics statement

The studies involving human participants were reviewed and approved by Local Ethics Committee for Research on Human Beings (opinion no. 2,776,490). The patients/participants provided their written informed consent to participate in this study.

## Author contributions

AA conceived and planned the experiments. RS and JB carried out the experiments. JB, LA, and PD contributed to the interpretation of the results. JB took the lead in writing the manuscript. All authors provided critical feedback and helped shape the research, analysis, and manuscript.

## Conflict of interest

The authors declare that the research was conducted in the absence of any commercial or financial relationships that could be construed as a potential conflict of interest.

## Publisher’s note

All claims expressed in this article are solely those of the authors and do not necessarily represent those of their affiliated organizations, or those of the publisher, the editors and the reviewers. Any product that may be evaluated in this article, or claim that may be made by its manufacturer, is not guaranteed or endorsed by the publisher.
